# Hierarchical stratification of the factors related to exertional dyspnoea and exercise intolerance in male COPD patients

**DOI:** 10.1080/07853890.2022.2135018

**Published:** 2022-10-31

**Authors:** Ming-Lung Chuang

**Affiliations:** a Department of Internal Medicine, Division of Pulmonary Medicine, Chung Shan Medical University Hospital, Taichung, ROC; b School of Medicine, Chung Shan Medical University, Taichung, ROC

**Keywords:** Exertional dyspnoea, incremental exercise test, diffusing capacity, air trapping, exercise capacity, cholesterol

## Abstract

**Background:**

The order and extent of interactions across the factors affecting exertional dyspnoea (ED) and exercise intolerance (EI) in patients with chronic obstructive pulmonary disease (COPD) are not clear. We hypothesized that lung and non-lung variables were the primary variables, ED was the secondary variable and EI was the tertiary variable.

**Methods:**

Data on demographics, blood tests, cardiac imaging, lung function tests and invasive dead space fractions (V_D_/V_T_) during incremental exercise test of 46 male COPD subjects were obtained. These variables were categorized by factor analysis and pair-wise correlation analysis was conducted. The best factor of each category was selected and then multivariate regression was conducted.

**Results:**

Peak tidal inspiratory flow (V_T_/T_Ipeak_), V_D_/V_Tpeak_ and tidal lung expansion capability, and resting diffusing capacity of the lungs (D_L_CO)% predicted were the primary pulmonary factors most related to ED, whereas body mass index (BMI), haemoglobin and cholesterol levels were the primary non-pulmonary factors. In multivariate regression analysis, V_T_/T_Ipeak_, V_D_/V_Tpeak_ and D_L_CO% were the primary factors most related to ED (*r*^2^ = 0.69); ED was most related to EI (*r* = −0.74 to −0.83).

**Conclusion:**

Using hierarchical stratification and statistical methods may improve understanding of the pathophysiology of ED and EI in patients with COPD.

**KEY MESSAGES**
- The pathophysiology of exertional dyspnoea (ED) and exercise intolerance (EI) in chronic obstructive pulmonary disease (COPD) is complex. The order and extent of interactions across factors are not clear. In multivariate regression analysis, we found that tidal inspiratory flow, dead space fraction and resting diffusing capacity of the lungs % but not the non-pulmonary factors affected ED.
- Using correlation coefficients, we further found that ED was the secondary variable and EI was the tertiary variable.
- Hierarchical stratification of the important factors associated with ED and EI in patients with COPD clarifies their relationships and could be incorporated into management programmes and outcome studies for these patients.

## Introduction

Exertional dyspnoea (ED) and exercise intolerance (EI) can seriously affect the physical activity, quality of life, and survival of patients with chronic obstructive pulmonary disease (COPD) [1,2]. Hence, understanding the mechanisms underlying ED and EI could help manage these patients.

Measurement of ED is a unique dimension and is important in evaluation of patients with COPD [3]. Subsequently, the pathophysiology of ED in COPD has evolved and was reported to be caused by an increase in inspiratory neural drive (chemostimulation) and abnormal dynamic respiratory mechanics and cardiovascular responses [4]. However, the reasons are complex and it is not clear in which order and to what extent these factors are pertinent to ED and EI and how they interact with each other. Nevertheless, measuring ventilatory mechanics requires the insertion of a diaphragmatic electromyography electrode catheter with oesophageal and gastric balloons [4]. The methods are invasive and sophisticated but are not practical for general practice.

Non-invasive methods to evaluate ED and EI would also be beneficial. A previous study using demographic data and lung physiology reported that forced vital capacity % (FVC%) predicted and forced expired volume in one second (FEV_1_)/FVC ratio explained ED with a power of only 13% [5]. Given that peak dead space fraction (V_D_/V_Tpeak_) and diffusing capacity of the lungs (D_L_CO) are the key mechanisms for ED in patients with COPD [6,7], it seems reasonable to include more factors when evaluating ED and EI. However, the use of multiple measures inflates the probability of a type I error [8]. Factor analysis techniques reduce multiple outcome measures to a lesser number of orthogonal dimensions [8,9].

In addition, in general, lung function is impaired to some extent before symptoms emerge, followed by EI and reduced quality of life, and eventually death [10]. This process is similar to the progression from pathobiology, pathophysiology and symptomatology to acute exacerbations of COPD [1] and is also similar to using different dimensions in development of survival analysis in patients with COPD [11] and in patients with COPD and chronic heart failure [12]. Hence, we hypothesized that hierarchical stratification combined regression analysis in variables of different categories is a reasonable and simple method to evaluate the pathophysiology of ED and EI.

The aim of this study was to establish the pathophysiology of ED and EI using correlation analysis in variables of different categories and hierarchical stratification, accordingly.

## Methods

### Study design

**Figure 1.**
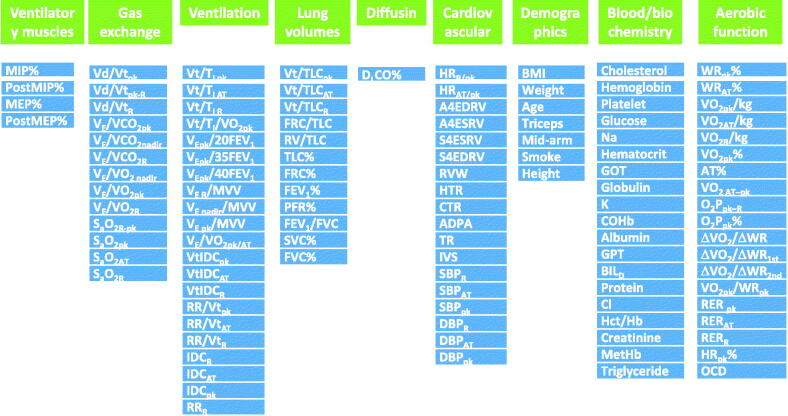
A total of 116 variables of interest in nine categories used to correlate with exertional dyspnoea (slope of Borg score and oxygen uptake). % = % predicted, subscribed R, AT, pk, R-pk or pk-R = variables measured at rest, anaerobic threshold, peak exercise, gradient between at rest and at peak exercise or vice versa, post: measured after exercise; ADPA: the diameter of anterior descending pulmonary artery in mm; A4EDRV: apical four-chamber view end-diastolic right ventricle area in cm^2^; A4ESRV: apical four-chamber view end-systolic right ventricle area in cm^2^; BIL_D_: direct bilirubin; BMI: body mass index; COHb: carboxyhaemoglobin; CTR: cardiac-thoracic ratio; Δ: delta = change; DBP: diastolic blood pressure; D_L_CO: diffusing capacity for carbon monoxide; FEV_1_: forced expired volume in one second; 20FEV_1_ = 20 × FEV_1_+20; FRC: functional residual capacity; FVC: forced vital capacity; GOT: aspartate aminotransferase; GPT: alanine aminotransferase; Hct/Hb: haematocrit/haemoglobin; HR: heart rate; HR_R/pk_: heart rate at rest and at peak exercise ratio; HR_AT/pk_: ratio of heart rate at and at peak exercise; HTR: Hila-thoracic ratio; IDC: inspiratory duty cycle; IVS: number of patients with paroxysmal intraventricular septum; MEP: maximum expiratory pressure; MetHb: methaemoglobin; Mid-arm: the mid-arm circumference in cm; MIP: maximum inspiratory pressure; MVV: maximum voluntary ventilation; OCD: oxygen-cost diagram; O_2_P: oxygen pulse; PFR: peak flow rate; pk/AT: ratio of the given values at peak and anaerobic threshold; RER: respiratory exchange ratio: V’CO_2_/V’O_2_; RR: respiratory rate; RV: residual volume; RVW: right ventricle free wall thickness in mm; S4EDRV: subcostal four-chamber view end-diastolic right ventricle area in cm^2^; S4ESRV: subcostal four-chamber view end-systolic right ventricle area in cm^2^; S_a_O_2_: arterial oxyhaemoglobin saturation; SBP: systolic blood pressure; SVC: slow vital capacity; Smoke: cigarette consumption in pack-years; T_I_: inspiratory time in second; TLC: total lung capacity; TR: number of patients with tricuspid valve regurgitation; Triceps: the thickness of triceps in mm; Vd/Vt: ratio of dead space and tidal volume: dead space fraction; V_E_: minute ventilation; V_E_/VCO_2_: ventilatory equivalent for CO_2_ output; VO_2_: Oxygen uptake; ΔVO_2_/ΔWR_1st and 2nd_: the first and 2^nd^ slopes of change in VO_2_ and work rate; V_T_: tidal volume; V_T_/T_I_: ratio of tidal volume and inspiratory time: mean inspiratory flow rate; V_T_/TLC: ratio of tidal volume and total lung capacity; WR: work rate.

**Figure 2.**
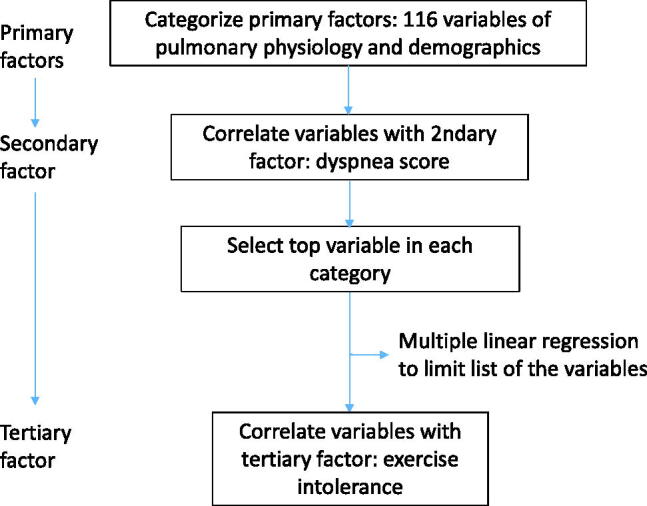
Flow chart illustrating the data processing steps.

We conducted this observational cross-sectional study to identify the most important factors affecting ED and EI using correlation analysis in different categories (Figure 1). The categories were established based on the previous reports using factor analysis [8,9]. We also used hierarchical stratification of these factors according to the power of correlation to evaluate the pathophysiology in subjects with COPD at our institution. This approach is based on the notion of the process of disease progression reported by Hurst and Wedzicha from pathobiology, pathophysiology and symptomatology to acute exacerbations of COPD [1] and by Gosker et al. from organ dysfunction (primary and secondary factors) to health status (tertiary factor) and survival outcomes (quaternary factor) [12]. Thus, the physiology of the heart and lung, demographics and biochemistry are considered to be the primary pulmonary and non-pulmonary factors, with symptoms as the secondary factors and EI as the tertiary factor [1,10]. Multiple linear regression was used when appropriate to select the most important factors. A flow chart illustrating the data processing steps is shown in Figure 2. Signed informed consent was obtained from each participant. The local Institutional Review Board of Chung Shan Medical University Hospital (CS19014) approved this study. This study was conducted in compliance with the Declaration of Helsinki.

### Subjects

We enrolled subjects with COPD aged ≥40 years with *a* ≥ 10 pack-year history of cigarette smoking. The diagnosis of COPD was based on the Global Initiative for Chronic Lung Disease (GOLD) criteria [13]. The FEV_1_/FVC was <0.7 [13]. The exclusion criteria were subjects with a body mass index (BMI) ≤18 or ≥32 kg·m^−2^, and those with uncontrolled diabetes mellitus, uncontrolled hypertension, moderate and severe anaemia (haemoglobin <10 g·dL^−1^ in males), cardiovascular, haematological, metabolic or neuromuscular diseases, as these factors may confound exercise performance. Subjects with acute illnesses in the recent 1 month were also excluded. The subjects with a BMI ≥32 kg·m^−2^ were excluded as this is considered to indicate severe obesity, and the pathophysiology of ED is different between obese and non-obese patients with COPD [14]. As few female subjects meet the criteria of COPD in Taiwan [15], female subjects were not included in this study.

### Measurements

Demographic data were collected. Leisure activity was coded 1–4 according to hours of activity per week: 1= ≤1 h; 2 = 1–3 h; 3 = 3–6 h; 4= >6 h [16]. Functional daily activity was assessed with oxygen-cost diagram (OCD) score [17].

Blood cell and biochemical analyses were conducted, and chest radiography was obtained within 1 month before commencement of the study. The hila-thoracic ratio (HTR), cardiac-thoracic ratio (CTR) and the diameter of anterior descending pulmonary artery (ADPA) on the standing posterior–anterior chest radiograph were measured [18]. Two-dimensional echocardiography was performed with parasternal, apical and subcostal studies by an experienced cardiologist who was unaware of the clinical data, lung function or cardiopulmonary exercise test reports [19,20].

Pre-test preparation and short-acting and long-acting beta bronchodilators administered were followed the standard rule as reported previously [9,21]. FEV_1_, FVC, total lung capacity (TLC), residual volume (RV) and diffusing capacity for carbon monoxide (D_L_CO) were measured, respectively, in accordance with the currently recommended standards [22,23]. The best of three technically satisfactory readings was used. All of the spirometry, static lung volume and D_L_CO data were obtained before inhaling 400 µg of fenoterol HCl. Post-dose spirometry was obtained 15 min after inhaling 400 µg of fenoterol HCl. Predicted normal values of spirometry, lung volumes and D_L_CO were taken from our previous studies so that the reports were consistent [9,21].

Maximum inspiratory pressure (MIP) at the mouth indicating inspiratory muscle strength was measured [3] at RV with a nose clip in place. A forceful inspiratory manoeuvre leading to a sustained maximal effort for the best 1–3 s was measured and followed by natural release upon fatigue [24]. Maximum expiratory pressure (MEP) indicating expiratory muscle strength at the mouth was measured at TLC. Predicted normal values of MIP and MEP were taken from Ruppel [25].

#### Cardiopulmonary exercise testing

Brachial artery catheterization was established in the non-dominant arm of each subject before exercise testing with pulmonary gas exchange measures. The subjects rested for 2 min, followed by unloaded and then incremental loaded exercise for 10 ± 2 min to limited symptoms. V′O_2_ (mL/min in STPD), CO_2_ output (V′CO_2_) (mL/min in STPD) and minute ventilation (V′_E_ in BTPS) and oxyhaemoglobin saturation (S_P_O_2_, %) were measured. Data of the last 15 s of each loaded stage were averaged and reported [26]. The modified Borg category scale used to rate dyspnoea perception and blood pressure were measured every 2 min during the test [27]. To scale ED, the ratio of change in Borg score and change in V′O_2_ (ΔBorg/ΔV′O_2_) was generated from the start of loading to peak exercise using linear regression. The rationale of using linear regression for this purpose was that the relationship between ΔBorg and ΔV′O_2_ during this period was quasi-linear even though it was curvilinear between the rest and peak exercise [28] and Borg score versus work rate was nearly linear in patients with COPD while it was curvilinear in normal subjects [6,29]. Predicted normal values of maximum V′O_2_ and watts were taken from Wasserman [30]. Dyspnoea index and breathing reserve were defined as follows:

(1) $$\text{Dyspnea index}=V{’}_{\text{Epeak}}/\text{maximum voluntary ventilation }\left(\text{MVV}\right)$$

(2) $$\text{Breathing reserve}=1-V{’}_{\text{Epeak}}/\text{MVV}$$

Blood samples were drawn and heparinized for each subject at peak exercise. The sample was immediately placed on ice and then analyzed for PCO_2_ with normal body temperature correction to calculate V_D_/V_Tpeak_ using the standard Bohr’s formula as follows [31].

(3) $$V_{D}/V_{\text{Tpeak}}=(P_{a}{\text{CO}}_{2}-P_{\bar{E}}{\text{CO}}_{2})/P_{a}{\text{CO}}_{2}-V_{D}m/V_{T}$$

where P_a_CO_2_ was arterial partial pressure of CO_2_, $P_{\bar{E}}$ CO_2_ was mixed expired PCO_2_ = V′CO_2_/V′_E_ × (P_B_ − 47 mm Hg), and P_B_ was barometric pressure in mm Hg measured daily and V_D_m was the dead space of the mouth piece and pneumotachograph according to the manufacturer’s instructions.

### Statistical analysis

Data were freely accessed by the investigators. Data were summarized as mean ± standard deviation. Pearson’s correlation coefficients were used when appropriate for quantifying the pair-wise relationships among the interested variables. Correlation analysis was also conducted in subsequent *post hoc* analyses when indicated. Hierarchical grouping the most important factors related to ED and exercise capacity was conducted. Multiple linear regression analysis was performed when indicated. All statistical analyses were performed using NCSS statistical software (NCSS 9, NCSS, LLC., Kaysville, UT, USA). Statistical significance was set at *p* < 0.05.

## Results

A total of 46 male subjects with COPD were enrolled (mean age 65.2 ± 5.8 years) (Table 1). Most of the participants had moderate to severe COPD and had obstructive ventilatory limitation (V’_Epeak_/MVV 116.4 ± 45.8%), mild hypercapnia and hypoxaemia at peak exercise (Tables 1 and 2). The modified Borg dyspnoea score was 8.6 ± 1.9 at peak exercise and 34 subjects had dyspnoea as the limiting symptom (i.e. 74% of all subjects).

**Table 1.** Demographic data, chest radiography, echocardiography and lung function data.

	*n*	Mean	SD
Age, year	46	65.2	5.8
Body mass index, kg/m^2^	46	22.12	3.53
Cigarette consumption, pack⋅year	46	42.3	19.2
Oxygen-cost diagram, cm	46	7.0	1.4
Haemoglobin, g/dL	46	14.8	1.5
Hila-thoracic ratio	44	0.36	0.04
Cardiac-thoracic ratio	41	0.44	0.06
Anterior descending pulmonary artery, cm	46	1.62	0.33
Apical 4* EDRV, cm^2^	42	13.5	3.7
Total lung capacity, TLC pred, %	46	135	21
Inspiratory capacity pred, %	46	90	26
RV/TLC, %	46	58	9
D_L_COpred, %	45	69	22
Forced vital capacity, FVC pred, %	46	81	21
FEV_1_pred, %	46	50	19
GOLD, I, II, II, IV, *n* (%) =	3 (7), 18 (39), 19 (41), 6 (13)		
FEV_1_/FVC, %	46	49	13
Maximal inspiratory pressure, %	43	63.8	17.2
Maximum expiratory pressure, %	43	51.6	11.2

D_L_CO: the diffusion capacity of the lungs for carbon monoxide; FEV_1_: forced expiratory volume in one second; GOLD: global initiative for chronic obstructive lung disease.

Leisure activity: coded 1–4 according to hours of activity per week: 1 = ≤1 h; 2 = 1–3 h; 3 = 3–6 h; 4 = >6 h, Anterior descending pulmonary artery of the right lung ≥1.8 cm indicating pulmonary hypertension, *apical four chamber view, end-diastolic right ventricle area (EDRV)>15 cm [2,21,22].

The data were reported in part recently [34].

**Table 2.** Pulmonary gas exchange at peak exercise.

Variables	*n*	Mean	SD
Oxygen uptake, L/min/kg	46	17.9	5.4
Heart rate, beat/min	46	133.2	20.4
S_P_O_2_, %	44	91.0	5.8
P_a_CO_2_, mm Hg	44	46.1	7.8
V_D_/V_T_	43	0.44	0.10
P_a-ET_CO_2,_ mm Hg	44	−0.55	5.04
Ventilatory equivalent for CO_2nadir_	46	35.0	6.9
Minute ventilation, L/min	46	38.6	12.3
Minute ventilation/MVV, %	46	116.4	35.8
Borg score, A.U.	46	8.6	1.9
ΔBorg/ΔV′O_2_, A.U./L	46	9.00	3.72

A − aDO_2_: alveolar arterial oxygen pressure gradient; A.U.: absolute unit; Δ: change; MVV: maximal voluntary ventilation; P_a-ET_CO_2_: arterial and end-tidal CO_2_ pressure gradient; S_P_O_2_: oxyhaemoglobin saturation measured by pulse oximetry; V_D_/V_T_: dead space and tidal volume ratio.

**Figure 3.**
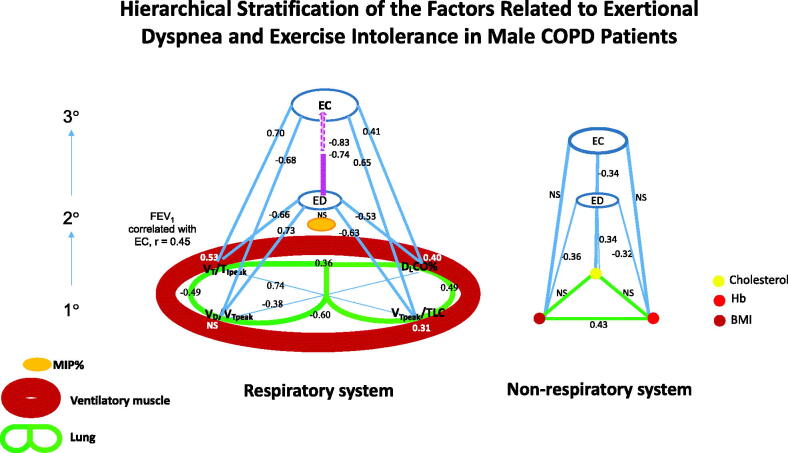
Hierarchical stratification of the pathophysiology of exertional dyspnoea and exercise intolerance (from primary (1°) to secondary (2°) to tertiary(3°)). Respiratory system (*left Panel*): the bottom (large circles indicating the lungs) indicates the relationships across the four primary pulmonary factors (PPFs); the large oval shape represents ventilatory muscle and the solid oval shape represents MIP%; the oval shape marked with ED i.e. exertional dyspnoea below the top oval shape is correlated with the PPFs; the top oval shape marked with EC i.e. exercise capacity is correlated with the PPFs and EC includes work rate at peak exercise % predicted (WR_peak_%) and oxygen uptake per kg at peak exercise (V′O_2peak_/kg). MIP%: maximum inspiratory pressure % predicted. Numbers indicate significant correlation coefficients; Numbers on the large oval shape indicate correlation coefficients between the PPFs and MIP%; NS indicates not significant. V_T_/T_Ipeak_: tidal inspiratory flow; D_L_CO%: diffusing capacity of lung % predicted; V_Tpeak_/TLC: peak tidal lung expansibility; V_D_/V_Tpeak_: peak dead space fraction. The correlation coefficients of ED with WR_peak_% = -0.83; of ED with V′O_2_/kg _peak_ = -0.74. Non-respiratory system (*right panel*): the bottom triangle indicates the relationships across the three primary non-pulmonary factors. The top circle indicates the cholesterol level; the right circle indicates haemoglobin (Hb) level; the left circle indicates body mass index (BMI).

ΔBorg/ΔV’O_2_ – an ED marker was successfully obtained in all participants (Table 2). Correlations between ΔBorg/ΔV’O_2_ and 116 variables of interest in nine categories were analysed (Supplementary table). The strongest variables in each category were identified. Peak mean tidal inspiratory flow (i.e. tidal volume divided by inspiratory time, V_T_/T_Ipeak_), tidal lung expansion capability (V_Tpeak_/TLC) and V_D_/V_Tpeak_, and D_L_CO% were the four primary pulmonary physiology factors most strongly correlated with ED – the secondary factor (Figure 3, left panel, |*r*| = 0.53–0.73, *p* = 0.0005– <0.0001) and with each other (|*r*|=0.36–0.74). Non-pulmonary primary factors including BMI, haemoglobin and serum cholesterol levels were mildly related to ΔBorg/ΔV’O_2_ (Figure 3, right panel, |*r*| = 0.32–0.36, all *p* < 0.05). Ventilatory muscle and cardiovascular categories were not correlated with ΔBorg/ΔV′O_2_. Correlations of the four primary pulmonary factors with exercise capacity – the tertiary factor (|*r*| = 0.41–0.70) were weaker than those with ED – the secondary factor (|*r*| = 0.53–0.73). However, ΔBorg/ΔV′O_2_ was even higher in relation to V′O_2peak_/kg and work _peak_% (*r*= −0.74 to –0.83, both *p* < 0.0001) than the primary pulmonary and non-pulmonary factors (|*r*| = 0.41 − 0.70, *p* < 0.01− <0.0001 and −0.04 to -0.34, p NS− <0.05). After hierarchical stratification the pathophysiology of ED and EI was established (Figure 3). In multiple linear regression analysis for ED with the seven variables, only V_T_/T_Ipeak_ (*p* = 0.005), V_D_/V_Tpeak_ (*p* = 0.03) and D_L_CO% (*p* = 0.04) were selected (Table 3, adjusted *r*^2^=0.69, *p* < 0.0001).

**Table 3.** Multiple linear regression analysis of exertional dyspnoea throughout the course of peak exercise (ΔBorg/ΔV’O_2_) in male patients with chronic obstructive pulmonary disease.

Exertional dyspnoea equation	Adjusted *r*^2^
15.03 (±5.99) −3.36 × V_T_/T_I peak_ (±1.12) + 10.61 × V_D_/V_T peak_ (±4.75) − 4.60 × D_L_CO% (±2.20)	0.69

Δ: change; Borg: Borg score; V′O_2_: oxygen uptake; V_T_/T_I peak_: tidal volume in litres divided by inspiratory time in seconds at peak exercise; V_D_/V_T_: dead space fraction; D_L_CO: diffusing capacity for carbon monoxide % predicted; (±SE): standard errors. Intercept, *p* = 0.01, V_T_/T_I peak_, *p* = 0.005, V_D_/V_T peak_, *p* = 0.03, D_L_CO%, *p* = 0.04, overall, *p* < 0.0001.

Table 4 in subsequent *post hoc* analyses, revealed the top four primary pulmonary factors relating to ED and their correlations. V_T_/T_Ipeak_ was associated with MIP% and spirometry (|*r*|=0.30–0.51), and negatively correlated with functional residual capacity (FRC)% and RV/TLC (*r*= −0.32 to -0.58). V_T_/T_Ipeak_/V’O_2peak_ was positively related to ΔBorg/ΔV′O_2_ (*r* = 0.38, *p* = 0.01). V_Tpeak_/TLC was positively related to MIP%, inspiratory capacity (IC)%, IC/TLC, inspiratory reserve volume (IRV)% and negatively to RV%. D_L_CO% was weakly related to FEV_1_% and FEV_1_/FVC and moderately related to RV/TLC. V_D_/V_Tpeak_ was inversely associated with BMI (*r*= −0.30, *p* = 0.047) and positively related to RV/TLC (*r* = 0.44) and P_a_CO_2_ and its derivatives (*r* = 0.24 − 0.71).

**Table 4.** Correlations of the top four primary pulmonary factors with variables of interest.

	*r*	*p*
**V_T_/T_Ipeak_**		
MIP%	0.53	0.0006
Spirometry: FVC% predicted	0.35	0.016
FEV_1_% predicted	0.51	0.0003
FEV_1_/FVC	0.3	0.04
Slow vital capacity % predicted	0.37	0.01
Peak expired flow rate % predicted	0.42	0.0035
Lung volumes: RV/TLC	^−^0.58	<0.0001
FRC % predicted	^−^0.32	0.03
V’_E peak_	0.95	<0.0001
**V_Tpeak_/TLC**		
IC% predicted	0.34	0.02
IRV% predicted	0.36	0.01
IC/TLC	0.52	0.0002
RV% predicted	^−^0.41	0.005
ERV% predicted	0.001	NS
**D_L_CO% predicted**		
RV/TLC	^−^0.42	0.004
FEV_1_% predicted	0.34	0.02
FEV_1_/FVC	0.35	0.02
**V_D_/V_T peak_**		
BMI, kg/m^2^	^−^0.30	0.047
RV/TLC	0.44	0.003
Slow vital capacity% predicted	^−^0.34	0.03
P_a_CO_2_, mm Hg	0.34	0.02
V′_E_/V′CO_2 nadir_	0.62	<0.0001
P_a−ET_CO_2_, mm Hg	0.71	<0.0001
P_a_O_2_, mm Hg	^−^0.15	NS
S_a_O_2_, %	^−^0.14	NS
A − aDO_2_, mm Hg	^−^0.00	NS
**MIP% predicted**		
Height, cm	0.41	0.01
Weight, kg	0.44	0.005
BMI, kg/m^2^	0.31	0.06
Albumin, g/dL	0.39	0.03
Smoke, pack-years		NS
FVC% predicted		NS
FEV_1_% predicted		NS
FEV_1_/FVC		NS
TLC% predicted		NS
RV/TLC		NS
IC% predicted	0.36	0.02
IC/TLC	0.48	0.002
IRV % predicted	0.37	0.02
IRV/TLC	0.49	0.002
ERV % predicted	^−^0.46	0.003
ERV/TLC	^−^0.37	0.02
FRC% predicted	^−^0.36	0.03
FRC/TLC	^−^0.54	0.0004

BMI: body mass index; D_L_CO: diffusing capacity for carbon monoxide; ERV: expiratory reserve volume; FEV_1_: forced expired volume in one second, FVC: forced vital capacity; FRC: functional residual capacity; HR: heart rate; IC: inspiratory capacity; IRV: inspiratory reserve volume; MIP: maximum inspiratory pressure; P_a_CO_2_: arterial PCO_2_; P_a–ET_CO_2_: the gradient between P_a_CO_2_ and end-tidal PCO_2_; RV/TLC: residual volume and total lung capacity ratio; S_a_O_2_: arterial oxyhaemoglobin saturation; SVC: slow vital capacity; TLC: total lung capacity; V_D_/V_T peak_: dead space fraction at peak exercise measured with arterial blood; V′_E peak_: minute ventilation at peak exercise; V′_E_/V′CO_2_ or V′_E_/V′O_2_: ventilatory equivalent for CO_2_ output or oxygen uptake; V_T_: tidal volume; T_I peak_: inspiratory time in seconds.

## Discussion

In this study, we successfully used ΔBorg/ΔV’O_2_ to scale ED in all participants. Using correlation analysis and hierarchical stratification, we clarified the order and extent of the associations among factors related to ED and EI.

V_T_/T_Ipeak_, V_Tpeak_/TLC, D_L_CO_%_ and V_D_/V_Tpeak_ were the top four primary lung physiology factors most strongly related to ED; ED was the secondary factor and most strongly related to EI (the tertiary factor). V_D_/V_Tpeak_ was the best and second best primary factor related to ED in univariate and multiple linear regression analyses, respectively. Tidal lung expansibility was not selected in multiple regression analysis, probably due to collinearity with peak mean tidal inspiratory flow and dead space fraction and resting D_L_CO% (|*r*|=0.49 − 0.74). The primary non-pulmonary physiology factors were much weaker in comparison (|*r*|=0.32 − 0.36). ED in patients with COPD is reported due to increased inspiratory neural drive (chemostimulation), abnormal dynamic respiratory mechanics and cardiovascular responses [4]. In this study, peak mean tidal inspiratory flow was the most strongly related to ED, followed by peak dead space fraction and resting diffusing capacity (Figure 3, |*r*|=0.53 − 0.73). This algorithm can be integrated with a neurobiological model [32] to improve the understanding of the pathophysiology of ED and EI.

### Peak mean tidal inspiratory flow (V_T_/T_I_)

Patients with COPD usually complain that ‘I cannot get air in’ rather than ‘My air goes out too slowly’ [33]. This may be due to the factor of V_T_/T_Ipeak_. V_T_/T_Ipeak_ was associated with MIP% and spirometry, and negatively correlated with trapped lung volumes (Table 4). Notably, when V_T_/T_Ipeak_ is normalized with V′O_2peak_, V_T_/T_Ipeak_/V′O_2peak_ refers to the mean tidal inspiratory flow at the corresponding V′O_2_, and the higher the value, the higher the energy cost for ventilation when consuming a commensurate metabolism, and was also positively related to ΔBorg/ΔV′O_2peak_ (*r* = 0.38). In addition, V_T_/T_Ipeak_ was highly correlated with minute ventilation (Table 4, *r* = 0.95) as they are both closely mathematically related;${\dot{V}}_{E}$ = 60 × V_T_/(T_I_ + T_E_) where T_E_ is expiratory time. Hence, V_T_/T_Ipeak_/V′O_2peak_ must be highly related to V′_E_/V′O_2peak_, a marker of inefficient ventilation or exercise hyperventilation. Notably, V_T_/T_Ipeak_ is the mean inspiratory flow measured at peak exercise, whereas peak inspiratory flow is the maximal flow rate obtained during an inspiratory manoeuvre. Peak inspiratory flow is related to maximal inspiratory pressure, and it has been shown to be a useful marker for how to select an appropriate inhaler device and clinical outcomes such as days to all-cause admission and COPD readmission [34].

### Tidal lung expansion capability and MIP%

V_Tpeak_/TLC represents tidal lung expandability at peak exercise. It indicates increased tidal volume and encroaches on IC%, IRV% and ERV% when exercising, and it is an inverse marker for dynamic hyperinflation in patients with COPD [35]. In this study, V_Tpeak_/TLC was positively related to MIP%, IC%, IC/TLC and IRV%, and negatively to RV% (Table 4). These findings are consistent with inspiratory muscles expanding the thoracic cage, thereby encroaching on IC% and IRV%. They are also supported by a study in which MIP was increased and RV and its relatives were decreased in patients with severe emphysema after lung volume reduction surgery [36].

Mahler and Harver reported that ventilatory muscle strength provides another unique dimension relating to the status of patients with COPD that was independent of both dyspnoea ratings and spirometry [3]. In this study, MIP% was mildly to moderately related to V_T_/T_Ipeak_, V_Tpeak_/TLC and D_L_CO% but not to V_D_/V_Tpeak_ suggesting that inspiratory muscles strike ventilation and thus affect V_T_/T_Ipeak_, V_Tpeak_/TLC and D_L_CO%, whereas V_D_/V_Tpeak_ is a unique dimension that is different from the aforementioned pulmonary physiological variables.

### Diffusion capacity of lung

D_L_CO was weakly related to severity of COPD (i.e. FEV_1_%) and moderately related to air trapping (Table 4). It is compatible with that D_L_CO is a different category from lung volumes or airflow [9]. Moreover, diffusion capacity of lung is a specific but insensitive predictor of abnormal gas exchange during exercise [37]. D_L_CO% was related to an indirect ED marker (Medical Research Council score, *r*^2^=0.18) [7]. Nevertheless, D_L_CO% was still an independent factor when correlating with ΔBorg/ΔV’O_2_ (Figure 3 and Table 3), which mimics a direct ED marker – Borg/V’O_2_% at peak exercise [7].

### Dead space fraction of lung and its contributors

A previous study demonstrated that V_D_/V_Tpeak_ was related to peak Borg/V′O_2_% with a power of 10% [7]. In this study, V_D_/V_Tpeak_ was strongly related to ΔBorg/ΔV′sO_2_ in univariate regression analysis (Figure 3, *r* = 0.73) and was also significant in multivariate regression analysis (Table 3). However, V_D_/V_Tpeak_ was obtained using invasive methods in this study.

V_D_/V_T_ at rest and at peak exercise was also shown to contribute to D_L_CO% in a previous report (*r*= −0.55 and −0.40)[7], and V_D_/V_Tpeak_ alone was related to D_L_CO% in this study (*r*= −0.38). V_D_/V_Tpeak_ also contributed to V_T_/T_Ipeak_ and V_Tpeak_/TLC (i.e. inverse dynamic hyperinflation) in this study (Figure 3), which is consistent with the report by Mahut et al. [7] In addition, V_D_/V_Tpeak_ was related to arterial oxygenation (*r*= −0.66) in a previous study [7], but not in the present study (Table 4). In contrast, V_D_/V_Tpeak_ was highly related to P_a_CO_2peak_ alone and its derivatives, including the gradient between P_a_CO_2_ and end-tidal PCO_2_ and V′_E_/V′CO_2nadir_ in this study (Table 4).

V_D_/V_Tpeak_ was inversely associated with BMI (*r*= −0.30, *p* = 0.047) and positively related to FRC – another marker of air trapping [38] and RV/TLC in this study (Table 4). These associations are consistent with multi-organ loss of tissue (MOLT) phenotype of COPD patients [39].

### Other factors

Haemoglobin level was related to ED in this study, probably because four subjects had mild anaemia (haemoglobin level 10.9–12.9 g/dL, Figure 3). However, the relationship was weak (Figure 3, *r*= −0.32) and was not selected when lung factors were considered. Mean serum cholesterol level remained significantly related to ED and exercise capacity even though participants with BMI ≥32 kg/m,^2^ coexisting cardiovascular disease, hyperlipidaemia and diabetes mellitus were excluded from this study. In addition, serum cholesterol level was not related to COPD [40], however, its level was variously and inversely related to hospitalization and death [41]. Recently, using lipidomics and statistical methods, unique lipid signature has been used to diagnose COPD independently of age, BMI, GOLD stages and FEV_1_%, but it has been shown to be associated with smoking pack–years [42]. However, in this study, serum cholesterol level was not related to smoking pack–years (p = NS). A possible link across inflammatory status, impaired metabolism, and lung function has been reported in patients with asthma COPD overlap [43]. Nevertheless, when physiological factors were considered simultaneously, BMI, haemoglobin and serum cholesterol levels were insignificantly related to ED.

### Hierarchical stratification

Taken together according to the power of correlation coefficient, the pathophysiology of ED (secondary factor) may be through the primary factor: (1) dynamic hyperinflation causing high tension of the diaphragm and thereby lowering MIP% and tidal inspiratory flow [44,45]; (2) high V_D_/V_Tpeak_ and impaired diffusing capacity of lung. ED was the best single predictive factor for EI (tertiary factor).

### Study limitations

Dynamic hyperinflation plays a major role in ED. However, it was not measured using repetitive inspiratory capacity manoeuvres in this study. Dynamic hyperinflation has been reported to be highly inversely related to V_Tpeak_/TLC in patients with COPD [35]. Thus, using V_Tpeak_/TLC may be more convenient to assess inverse dynamic hyperinflation instead of performing repetitive inspiratory capacity manoeuvres. In addition, it may be argued that measuring V_D_/V_Tpeak_ is invasive and redundant, as V′_E_/V′CO_2_ and V′_E_/V′O_2_, their nadir values, slopes and intercepts during exercise give a very good approximation of the 'wasted’ ventilation. V′_E_/V′CO_2nadir_ was reported to be strongly related to V_D_/V_T_ in a previous study [6,7] and this report (Table 4, *r* = 0.62 and *r* = 0.78 in a previous report [6]). However, V′_E_/V′CO_2nadir_ was more weakly correlated with ΔBorg/ΔV’O_2_ in comparison to V_D_/V_Tpeak_ (*r* = 0.38 versus 0.73). Although the markers of cardiovascular function were used to correlate with ED and exercise capacity in this study (Table 1 and Figure 1), they were not contributory even though these markers were measured at rest and during exercise.

### Clinical implication

ΔBorg/ΔV′O_2_ can be used to scale ED. A hierarchical approach to the pathophysiology of ED and EI is informative and educational. Understanding the factors related to ED and thereby EI is important for clinicians when managing patients with COPD.

## Conclusions

In hierarchical stratification of the relevant factors in subjects with COPD, mean tidal inspiratory flow and dead space fraction at peak exercise, and diffusing capacity of the lungs were the primary factors related to ED; ED was the best single secondary factor related to EI – the tertiary factor. The analysis may improve understanding of the pathophysiology of ED and EI in patients with COPD.

## Supplementary Material

Supplemental Material

## Data Availability

The raw data as the file ‘Supplement data’ was uploaded. M-L.C. confirms that the data supporting the findings of this study are available within the article and its supplementary materials.
